# Supplementary material for: The impact of vaccination to control COVID-19 burden in the United States: A simulation modeling approach
Source: PLoS One. 2021 Jul 14;16(7):e0254456. doi: 10.1371/journal.pone.0254456 (PMC8279349; doi:10.1371/journal.pone.0254456)
Supplement: S1 Data — (DOCX) [file pone.0254456.s001.docx]

**Supplementary Material**

Table of Contents

[Section A. Details of COVAM 3](#_Toc75964006)

[Overview of COVAM 3](#_Toc75964007)

[Figure s1. Progression of COVID-19 3](#_Toc75964008)

[Disease states 3](#_Toc75964009)

[Adherence to NPIs 4](#_Toc75964010)

[Parameter estimation 4](#_Toc75964011)

[Table s1. COVAM parameters specific to regions. 5](#_Toc75964012)

[Table s2. Input parameters of COVAM 6](#_Toc75964013)

[Section B. Model Validation Results 8](#_Toc75964014)

[Figure s2. Model validation results. 8](#_Toc75964015)

[Section C. Sensitivity Analyses 10](#_Toc75964016)

[Section C.1 Sensitivity analysis on the drop in adherence to nonpharmaceutical interventions after vaccination 10](#_Toc75964017)

[Table s3. Controllable spread date and number of cases on December 31, 2021 for different vaccination capacity scenarios when there is no drop in adherence to nonpharmaceutical interventions after vaccination (vaccine effectiveness 90%, vaccine coverage 50%) 10](#_Toc75964018)

[Table s4. Controllable spread date and number of cases on December 31, 2021 for different vaccination effectiveness scenarios when there is no drop in adherence to nonpharmaceutical interventions after vaccination (vaccine coverage 50%, vaccination capacity 0.25%) 12](#_Toc75964019)

[Figure s3. Impact of vaccine coverage on the number of confirmed cases in different regions for two different scenarios of adherence to nonpharmaceutical interventions when there is no drop in adherence after vaccination 14](#_Toc75964020)

[Figure s4. Impact of vaccination and adherence to nonpharmaceutical interventions on the number of cases over time when there is no drop in adherence after vaccination 17](#_Toc75964021)

[Section C.2 Sensitivity analysis on the baseline probability of testing 20](#_Toc75964022)

[Figure s5. Comparison of model predictions to actual NYC data when the baseline probability of testing is equal to 50% 20](#_Toc75964023)

[Table s5. Controllable spread date and number of cases on December 31, 2021 for different daily vaccination capacity scenarios when the baseline probability of testing is equal to 50% (vaccine effectiveness 90%, vaccine coverage 50%). 21](#_Toc75964024)

[Table s6. Controllable spread date and number of cases on December 31, 2021 for different daily vaccination effectiveness scenarios when the baseline probability of testing is equal to 50% (vaccine coverage 50%, vaccination capacity 0.25%). 22](#_Toc75964025)

[Figure s6. Impact of vaccine coverage on the number of confirmed cases in different regions for two different scenarios of adherence to nonpharmaceutical interventions when the baseline probability of testing is equal to 50% 23](#_Toc75964026)

[Figure s7. Impact of vaccination and adherence to nonpharmaceutical interventions on the number of cases over time when the baseline probability of testing is equal to 50%. 24](#_Toc75964027)

[Section C.3 Sensitivity analysis on the modeling of vaccination effect 25](#_Toc75964028)

[Figure s8. Comparison of model predictions to actual NYC data when vaccination effectiveness is modeled as a reduction in probability of becoming infected 25](#_Toc75964029)

[Table s7. Controllable spread date and number of cases on December 31, 2021 for different daily vaccination capacity scenarios 26](#_Toc75964030)

[Table s8. Controllable spread date and number of cases on December 31, 2021 for different vaccination effectiveness scenarios when vaccination effectiveness is modeled as a reduction in probability of becoming infected (vaccine coverage 50%, vaccination capacity 0.25%) 27](#_Toc75964031)

[Figure s9. Impact of vaccine coverage on the number of confirmed cases in different regions for two different scenarios of adherence to nonpharmaceutical interventions when vaccination effectiveness is modeled as a reduction in probability of becoming infected 28](#_Toc75964032)

[Figure s10. Impact of vaccination and adherence to nonpharmaceutical interventions on the number of cases over time when vaccination effectiveness is modeled as a reduction in probability of becoming infected 29](#_Toc75964033)

# Section A. Details of COVAM

## Overview of COVAM

COVAM is an agent-based simulation model in which agents represent the individuals in a community. At the beginning, no agents have immunity to COVID-19. In each time step, representing one day, agents may interact with other agents randomly where the number of close daily interactions dependent on the agent’s age and adherence to social distancing. The number of daily interactions decreases with patient age, with younger agents maintaining most of their daily interactions. COVID-19 exposure may occur if a contagious agent closely interacts with a susceptible agent. The model considers that some infectious individuals are never tested positive, a number of new infectious agents are imported into the region representing the case that there is movement among regions, and some asymptomatic individuals transmit the disease prior to showing symptoms. COVAM represents the effects of nonpharmaceutical interventions (NPIs) such as physical distancing measures and facial mask use by reducing the number of daily interactions. COVAM coded programmed using C++ programming language for flexibility and quicker computational times. Each simulation run lasts between several minutes and an hour on a standalone desktop PC that varies by scenario and population size. Full details of COVAM are published elsewhere (1) and briefly summarized here.

Figure s1. Progression of COVID-19 (adapted from Alagoz, et al 2020(1))


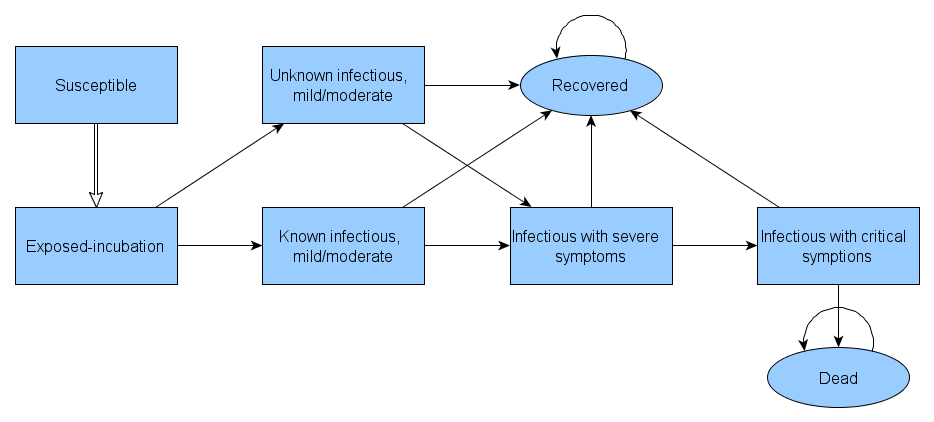


## Disease states

Agents may occupy one of the following eight disease states: Susceptible (S), Exposed-incubation (E), Infectious with mild to moderate symptoms that is detected (IM+), Infectious with mild to moderate symptoms that is undetected (IM-), Infectious with severe symptoms (IS), Infectious with symptoms requiring critical care (IC), Recovered (R), and Dead (D). If a susceptible agent is exposed, they will transition to the exposed-incubation phase (E), in which they will remain for on average five days. On average, during the last two days of an exposed-incubation agent may become infectious and expose others. The agent may then transition to an infectious with mild symptoms state, dependent on if they are tested (IM- or IM+).

Regardless of testing, agents remain infectious with mild symptoms for an average of six days. The agent may then transition to the recovered stay, or to severe symptoms (IS). Patients in the IS state are considered to be hospitalized and have severe symptoms including “dyspnea, hypoxia, or >50% lung involvement on imaging,” according to the CDC definition (1). The agent remains in the IS state for an average of 6 days. The agent may then again transition to the recovered state or to the IC state, denoting critical symptoms and requiring an ICU stay. An average of 46% of patients in the IC state will require use of a ventilator. Patients in the IC state remain in this state for an average of ten days before transitioning to either the recovery state or death state. Probability of recovering from mild, severe, or critical symptoms is dependent on agent age. Supplement Figure s1 illustrates the discrete time Markov chain representing patient disease state.

## Adherence to NPIs

COVAM uses a detailed representation of the varying adherence levels of the communities to NPIs. COVAM uses adherence to NPIs to represent several behaviors that reduce the transmissibility of SARS-CoV-2, including keeping at least 6-feet of distancing during person-to-person interactions, less frequent traveling, and wearing masks. Our primary source for the adherence to NPIs is distance traveled and maintenance of at least 6-foot distance during person-to-person interactions that relies on cell-phone data. Although COVAM has the ability to adjust this parameter on a daily basis, we changed the value of this parameter infrequently to prevent overfitting.

## Parameter estimation

Parameters used in COVAM are described in Supplement Tables 1 and 2. Parameters were primarily estimated from observational data and literature. In this model, we assume that agents in the IS and IC states cannot transmit COVID-19 to other agents as interactions with the population are cut off during a hospital stay. Given this assumption, the probability for transmission from mildly symptomatic patients to susceptible patients is a key driver of transmissibility in this model. To estimate this parameter, we find a probability that, along with other baseline parameters, will result in a theoretical R_0_ of 2.6. This R_0_ value was calculated using data from China early in the pandemic (2). The resultant probability of transmission is 0.0418, which we assume applies to asymptomatic and symptomatic agents regardless of testing status.

To estimate the adherence to NPIs over time, we used multiple sources that estimated mobility changes utilizing mobile phone data (3-5). We also use the change in mobility as a proxy for adherence to other behaviors that reduce the risk of transmission, such as increased hand hygiene and face mask use. Age-specific adherence to social distancing was estimated by calculating relative rates by age of other age-specific parameters and applying these rates to distancing and contacts.

Several parameters in COVAM that could not be estimated from observational data or literature were calibrated using observed number of confirmed cases over time. To prevent overfitting, we did not seek to exactly match the output of the model with observed data. We considered that testing availability changed over time as there was limited COVID-19 testing in the early days of pandemic. Similarly, we considered that the hospitalization rates have changed over time since compared to the early days of pandemic, the proportion of patients with a positive COVID-19 test result and are hospitalized over patients with a positive COVID-19 test result dropped over time.

Table s1. COVAM parameters specific to regions. (adapted from Alagoz, et al 2020 (1))

| Parameter | Value, by region | | | Source |
| --- | --- | --- | --- | --- |
|  | Dane County | Milwaukee Metro | New York City |  |
| Population size | 542,364 | 1,576,236 | 8,398,748 | US Census data (6) |
| Date of simulation start | March 4th, 2020 | March 4th, 2020 | March 4th, 2020 | -- |
| Number of exposed agents at start of simulation | 1 | 3 | 16 | Calibration |
| Number of daily imported exposed agents and dates of importation | 3 daily from Mar 5- 25, 2020; 1 daily onward | 6 daily from Mar 5-25, 2020; 3 daily onward | 160 daily from Mar 5-25, 2020; 32 daily onward | Calibration |
| Number of daily close contacts without social distancing | 10 | 10 | 20 | Calibration and literature (7, 8) |
| Adherence to social distancing, expressed as percentage drop in number of contacts | Mar 4 - 11, 2020: 0% Mar 12 - 25, 2020: linear increase from 0 - 91% Mar 26 - Apr 30, 2020: 80% May 1 - 13, 2020: 70%  May 14 - June 30, 2020: 60%  July 1 - Aug 31, 2020: 70%  Sep 1 - Nov 8, 2020: 65% Nov 9, 2020 – end of simulation: 70% | Mar 4 – 11, 2020: 0% Mar 12 - 22, 2020: linear increase from 0 - 60% Mar 23 - Apr 7, 2020: 60% Apr 8 - 12, 2020: 80% Apr 13 - May 13, 2020: 70% May 14 - 25, 2020: 60% May 26 - July 15, 2020: 70% July 16 - Aug 14, 2020: 65%  Aug 15 - Sep 29, 2020: 70%  Sep 30 - Oct 29, 2020: 60%  Oct 30 - Nov 8, 2020: 65%  Nov 9, 2020– end of simulation: 70% | Mar 4 - 11, 2020: 0% Mar 12 - 25, 2020: linear increase from 0 - 78% Mar 26 - Apr 17, 2020: 85%  Apr 18 - June 7, 2020: 90%  June 8 - July 1, 2020: 85%  July 2 - Aug 15, 2020: 90%  Aug 16 - Sep 14, 2020: 80%  Sep 15 - Nov 14, 2020: 85%  Nov 15 - Dec 5, 2020: 70%  Dec 6 - Dec 31, 2020: 85%  Jan 1, 2021 – end of simulation: 80% | Cellphone data and calibration (3-5) |
| Probability that agent is tested | Mar 4 - Apr 14, 2020: 75% Apr 15 - May 9, 2020: 80% May 10, 2020 - end: 90% | Mar 4 - Apr 14, 2020: 75% Apr 15 - May 9, 2020: 80% May 10, 2020 - end: 90% | Mar 4 - Apr 14, 2020: 75% Apr 15 - May 9, 2020: 80% May 10, 2020 - end: 90% | Literature and calibration (9, 10) |
| Age distribution (percentage) | 0-19y: 20.8% 20-44y: 46.5% 45-54y: 10.3% 55-64y: 10.2% 65-74y: 7.4% 75-84y: 3.3% 85y+: 1.5% | 0-19y: 28.6% 20-44y: 36.2% 45-54y: 13.7% 55-64y: 8.5% 65-74y: 6.6% 75-84y: 4.7% 85y+: 1.0% | 0-19y: 23.2% 20-44y: 38.6% 45-54y: 13.0% 55-64y: 11.7% 65-74y: 7.1% 75-84y: 3.4% 85y+: 1.9% | US Census data (6) |

Table s2. Input parameters of COVAM (adapted from Alagoz, et al 2020(1))

| Parameter | Mean Value | Source |
| --- | --- | --- |
| Progression | | |
| Incubation time between exposure and onset of symptoms | 5 days | CDC and literature (11-14) |
| Duration of mild symptoms | 6 days | CDC and literature (11, 15-17) |
| Duration of severe symptoms | 6 days | CDC and literature (11, 15-17) |
| Duration of critical symptoms | 10 days | CDC and literature (11, 15-18) |
| Baseline recovery probability after mild symptoms, by age | 0-19y: 98% 20-44y: 82% 45-54y: 75% 55-64y: 75% 65-74y: 64% 75-84y: 55% 85y+: 49% | CDC (19) |
| Changes in recovery probability after mild symptoms (i.e. 1- the baseline recovery probabilities are multiplied with this parameter so that the recovery probabilities are adjusted upwards) | Before Jun 1, 2020: 1  Jun 1 -Jul 15, 2020: 0.5  after Jul 15, 2020: 0.25 | Public Health Madison and Dane County (20) and calibration |
| Recovery probability after severe symptoms, by age | 0-19y: >99% 20-44y: 82% 45-54y: 68% 55-64y: 69% 65-74y: 63% 75-84y: 53% 85y+: 65% | CDC (19) |
| Recovery probability after critical symptoms, by age | 0-19y: >99% 20-44y: 95% 45-54y: 92% 55-64y: 75% 65-74y: 72% 75-84y: 64% 85y+: 9% | CDC and Wisconsin Department of Health Services (19, 21) |
| Probability of ventilator support for critical patient | 46% | Literature (12) |
| Transmission | | |
| Number of daily contacts without social distancing | 10 | Calibration and literature (7, 8) |
| Relative rate of daily contact changes by age, relative to 20-44y group | 0-19y: 85% 20-44y: 100% 45-54y: 94% 55-64y: 74% 65-74y: 46% 75-84y: 34% 85y+: 34% | Literature (7) |
| Relative rate of social distancing non-adherence changes by age, relative to 20-44y group | 0-19y: 100% 20-44y: 100% 45-54y: 100% 55-64y: 85% 65-74y: 61% 75-84y: 61% 85y+: 61% | CDC (22) |
| Number of days an exposed patient is contagious | 2 | Literature (23, 24) |
| Probability of transmission from exposed agent to susceptible agent in close contact | 0.0418 | Calibration and literature (2, 25, 26) |

# Section B. Model Validation Results

In this section, we present the updated results of the validation of COVAM. Model structure and all model inputs for Dane County, Milwaukee metro area, and NYC except vaccination and adherence to NPIs were fixed before July 31, 2020. The structure of the vaccination and vaccination-related inputs were fixed by December 15, 2020. Adherence to social distancing inputs were fixed by January 5, 2021 for Dane County and Milwaukee and by January 14, 2021 for NYC and the predictions of COVAM were compared against the reported number of confirmed cases over time after this date.

In these runs, we used the following settings for the model inputs: vaccination effectiveness is 90%, vaccination coverage is 50%, daily vaccination capacity is 0.1%, drop in adherence after vaccination is 20%, number of imported cases is 1, 3, and 16 for Dane County, Milwaukee, and NYC, respectively, and adherence to NPIs after February 1, 2021 is 70%, 70%, and 85%, for Dane County, Milwaukee, and NYC, respectively. The results in this section use the average values of 100 replications, providing steady estimates around the mean parameter values with small standard errors. Data on case numbers were obtained from the Wisconsin Department of Health Services and the NYC Department of Health (21, 27).

Figure s2. Model validation results. In each of the following figures, red dots represent the actual observed cumulative number of confirmed cases, the black solid line represents the model’s predictions, error bars around the black solid line represent 95% confidence intervals for the model’s predictions based on 100 replications, the green dotted line represents the date after which no model input parameter except adherence to NPIs was modified, and the blue dashed line represents the date after which no model input parameter was modified.

(a) Dane County

(b) Milwaukee

(c) NYC

# Section C. Sensitivity Analyses

We conducted a sensitivity analysis on several parameters. In this appendix, we describe these experiments.

## Section C.1 Sensitivity analysis on the drop in adherence to nonpharmaceutical interventions after vaccination

Our base-case assumes that there is a 20% drop in adherence to NPIs among vaccinated individuals regardless of whether they gain immunity or not after vaccination. In this sensitivity analysis, we did all experiments by assuming that there is no drop in adherence to NPIs.

Table s3. Controllable spread date and number of cases on December 31, 2021 for different vaccination capacity scenarios when there is no drop in adherence to nonpharmaceutical interventions after vaccination (vaccine effectiveness 90%, vaccine coverage 50%). Numbers in parentheses represent percent reduction relative to no vaccine.

| Dane County | | | | | | | | | | | | | | |  |
| --- | --- | --- | --- | --- | --- | --- | --- | --- | --- | --- | --- | --- | --- | --- | --- |
|  | 75% Adherence | | 70% Adherence | | | | 65% Adherence | | | 60% Adherence | | Dynamic Adherence | | |  |
| Vaccination capacity | Date | Number of cases | Date | | | Number of cases | Date | Number of cases | | Date | Number of cases | Date | Number of cases |  |  |
| No Vaccine | 27-Mar-2021 | 43,932 | 18-Jul-2021 | | | 51,784 | 17-Mar-2022 | 111,014 | | 29-Oct-2021 | 200,642 | After June 2022 | 79,285 |  |  |
| 0.05% | 22-Mar-2021 | 43,222  (2%) | 25-May-2021 | | | 48,016  (7%) | 20-Oct-2021 | 73,747  (34%) | | 14-Oct-2021 | 150,022  (25%) | 18-Mar-2022 | 72,088  (9%) |  |  |
| 0.1% | 18-Mar-2021 | 42,697  (3%) | 2-May-2021 | | | 46,110  (11%) | 4-Aug-2021 | 59,452  (46%) | | 17-Sep-2021 | 109,029  (46%) | 28-Oct-2021 | 64,654  (18%) |  |  |
| 0.25% | 12-Mar-2021 | 42,164  (4%) | 1-Apr-2021 | | | 43,374  (16%) | 4-May-2021 | 46,128  (58%) | | 16-Jun-2021 | 53,602  (73%) | 30-May-2021 | 46,579  (41%) |  |  |
| 0.5% | 27-Feb-2021 | 40,720  (7%) | 10-Mar-2021 | | | 41,514  (20%) | 26-Mar-2021 | 42,903  (61%) | | 13-Apr-2021 | 45,483  (77%) | 30-Mar-2021 | 42,547  (46%) |  |  |
| Milwaukee | | | | | | | | | | | | | | |  |
|  | 75% Adherence | | | | 70% Adherence | | 65% Adherence | | | 60% Adherence | | Dynamic Adherence | | |  |
| Vaccination capacity | Date | Number of cases | | Date | | Number of cases | Date | | Number of cases | Date | Number of cases | Date | Number of cases | |  |
| No Vaccine | 23-Mar-2021 | 167,626 | | 12-Jun-2021 | | 185,343 | 26-Mar-2022 | | 309,809 | 18-Nov-2021 | 567,311 | After June 2022 | 271,692 | |  |
| 0.05% | 18-Mar-2021 | 165,699  (1%) | | 7-May-2021 | | 177,194  (4%) | 19-Sep-2021 | | 230,229  (26%) | 20-Oct-2021 | 418,533  (26%) | 2-Feb-2022 | 252,269  (7%) | |  |
| 0.1% | 14-Mar-2021 | 164,239  (2%) | | 21-Apr-2021 | | 172,714  (7%) | 11-Jul-2021 | | 201,894  (35%) | 10-Sep-2021 | 312,232  (45%) | 2-Oct-2021 | 213,120  (22%) | |  |
| 0.25% | 6-Mar-2021 | 161,330  (4%) | | 27-Mar-2021 | | 165,781  (11%) | 28-Apr-2021 | | 175,693  (43%) | 7-Jun-2021 | 201,573  (64%) | 15-May-2021 | 173,455  (36%) | |  |
| 0.5% | 26-Feb-2021 | 158,587  (5%) | | 8-Mar-2021 | | 160,720  (13%) | 21-Mar-2021 | | 164,264  (47%) | 7-Apr-2021 | 170,621  (70%) | 26-Mar-2021 | 163,338  (40%) | |  |
| NYC | | | | | | | | | | | | | | |  |
|  | 90% Adherence | | | | 85% Adherence | | 80% Adherence | | | 75% Adherence | | Dynamic Adherence | | | |
| Vaccination capacity | Date | Number of cases | | Date | | Number of cases | Date | Number of cases | | Date | Number of cases | Date | Number of cases |  |  |
| No Vaccine | 22-Mar-2021 | 699,352 | | 27-Aug-2021 | | 1,110,070 | 4-Sep-2021 | 3,307,090 | | 21-Jun-2021 | 4,999,100 | After June 2022 | 1,308,990 |  |  |
| 0.05% | 19-Mar-2021 | 686,290  (2%) | | 28-Jun-2021 | | 987,825  (11%) | 20-Aug-2021 | 2,765,350  (16%) | | 20-Jun-2021 | 4,562,030  (9%) | After June 2022 | 1,274,190  (3%) |  |  |
| 0.1% | 17-Mar-2021 | 674,662  (4%) | | 31-May-2021 | | 911,821  (18%) | 3-Aug-2021 | 2,290,640  (31%) | | 19-Jun-2021 | 4,120,280  (18%) | 16-Feb-2022 | 1,193,630  (9%) |  |  |
| 0.25% | 11-Mar-2021 | 647,683  (7%) | | 23-Apr-2021 | | 786,153  (29%) | 16-Jun-2021 | 1,394,020  (58%) | | 11-Jun-2021 | 2,843,430  (43%) | 31-Jul-2021 | 865,510  (34%) |  |  |
| 0.5% | 4-Mar-2021 | 616,204  (12%) | | 27-Mar-2021 | | 687,376  (38%) | 30-Apr-2021 | 888,980  (73%) | | 6-Jun-2021 | 1,463,240  (71%) | 19-May-2021 | 696,766  (47%) |  |  |

Table s4. Controllable spread date and number of cases on December 31, 2021 for different vaccination effectiveness scenarios when there is no drop in adherence to nonpharmaceutical interventions after vaccination (vaccine coverage 50%, vaccination capacity 0.25%). Numbers in parentheses represent percent reduction in the number of cases relative to no vaccine.

| Dane County | | | | | | | | | | |
| --- | --- | --- | --- | --- | --- | --- | --- | --- | --- | --- |
|  | 75% Adherence | | 70% Adherence | | 65% Adherence | | 60% Adherence | | Dynamic Adherence | |
| Vaccine effectiveness | Date | Number of cases | Date | Number of cases | Date | Number of cases | Date | Number of cases | Date | Number of cases |
| No Vaccine | 27-Mar-2021 | 43,932 | 18-Jul-2021 | 51,784 | 17-Mar-2022 | 111,014 | 29-Oct-2021 | 200,642 | After June 2022 | 79,285 |
| 50% | 18-Feb-2021 | 39,660 (10%) | 26-Feb-2021 | 40,235 (22%) | 18-Mar-2021 | 41,456  (63%) | 4-Jun-2021 | 45,520 (77%) | 12-May-2021 | 44,345 (44%) |
| 75% | 10-Feb-2021 | 38,702 (12%) | 12-Feb-2021 | 38,905 (25%) | 17-Feb-2021 | 39,220  (65%) | 26-Feb-2021 | 39,792 (80%) | 22-Feb-2021 | 39,719 (50%) |
| 90% | 6-Feb-2021 | 38,288 (13%) | 8-Feb-2021 | 38,402 (26%) | 9-Feb-2021 | 38,560  (65%) | 12-Feb-2021 | 38,798 (81%) | 11-Feb-2021 | 38,774 (51%) |
| Milwaukee | | | | | | | | | | |
|  | 75% Adherence | | 70% Adherence | | 65% Adherence | | 60% Adherence | | Dynamic Adherence | |
| Vaccine effectiveness | Date | Number of cases | Date | Number of cases | Date | Number of cases | Date | Number of cases | Date | Number of cases |
| No Vaccine | 23-Mar-2021 | 167,626 | 12-Jun-2021 | 185,343 | 26-Mar-2022 | 309,809 | 18-Nov-2021 | 567,311 | After June 2022 | 271,692 |
| 50% | 17-Feb-2021 | 155,364 (7%) | 25-Feb-2021 | 156,941 (15%) | 13-Mar-2021 | 160,000  (48%) | 4-May-2021 | 168,546 (70%) | 14-Apr-2021 | 165,711 (39%) |
| 75% | 10-Feb-2021 | 152,524 (9%) | 12-Feb-2021 | 153,087 (17%) | 17-Feb-2021 | 153,968  (50%) | 23-Feb-2021 | 155,430 (73%) | 22-Feb-2021 | 155,239 (43%) |
| 90% | 5-Feb-2021 | 151,249 (10%) | 7-Feb-2021 | 151,565 (18%) | 9-Feb-2021 | 151,985  (51%) | 12-Feb-2021 | 152,620 (73%) | 10-Feb-2021 | 152,551 (44%) |
| NYC | | | | | | | | | | |
|  | 90% Adherence | | 85% Adherence | | 80% Adherence | | 75% Adherence | | Dynamic Adherence | |
| Vaccine effectiveness | Date | Number of cases | Date | Number of cases | Date | Number of cases | Date | Number of cases | Date | Number of cases |
| No Vaccine | 22-Mar-2021 | 699,352 | 27-Aug-2021 | 1,110,070 | 4-Sep-2021 | 3,307,090 | 21-Jun-2021 | 4,999,100 | After June 2022 | 1,308,990 |
| 50% | 25-Feb-2021 | 566,553 (19%) | 24-Mar-2021 | 612,820 (45%) | 13-Aug-2021 | 835,071  (75%) | 20-Oct-2021 | 2,107,950 (58%) | After June 2022 | 1,134,180 (13%) |
| 75% | 17-Feb-2021 | 530,075 (24%) | 2-Mar-2021 | 547,058 (51%) | 2-Apr-2021 | 590,913  (82%) | 8-Oct-2021 | 820,471 (84%) | 25-Nov-2021 | 683,294 (48%) |
| 90% | 14-Feb-2021 | 513,890 (27%) | 21-Feb-2021 | 523,333 (53%) | 7-Mar-2021 | 542,626  (84%) | 27-Apr-2021 | 599,724 (88%) | 27-Mar-2021 | 564,244 (57%) |

Figure s3. Impact of vaccine coverage on the number of confirmed cases in different regions for two different scenarios of adherence to nonpharmaceutical interventions when there is no drop in adherence after vaccination. In this figure, we assumed that vaccine effectiveness is equal to 90%, vaccine capacity is 0.25%/day.

a. Dane, adherence to NPIs is fixed at a level of 70%

b. Dane, adherence to NPIs is dynamic

c. Milwaukee, adherence to NPIs is fixed at a level of 70%

d. Milwaukee, adherence to NPIs is dynamic

e. NYC, adherence to NPIs is fixed at a level of 85%

f. NYC, adherence to NPIs is dynamic

Figure s4. Impact of vaccination and adherence to nonpharmaceutical interventions on the number of cases over time when there is no drop in adherence after vaccination. In these experiments, we assumed that vaccine effectiveness is 90%, vaccine coverage is 50%, and daily vaccination capacity is 0.25% for the vaccination scenario.

a. Dane, vaccination

b. Dane, No vaccination

c. Milwaukee, vaccination

d. Milwaukee, no vaccination

e. NYC, vaccination

f. NYC, no vaccination

## Section C.2 Sensitivity analysis on the baseline probability of testing

COVAM assumes that the probability of testing changes over time due to increasing testing capacity in the US. Our initial calibration estimated that the baseline probability of testing in the early days of pandemic was 75% however, it is possible that we overestimated this parameter since data from NYC show that one of every five residents tested positive for COVID-19 antibodies (28). We conducted a sensitivity analysis to address the impact of uncertainty in this parameter, in which we assumed that the probability of testing is 50% and calibrated the parameter on adherence to NPIs. We conducted this experiment only for NYC. We modified only adherence to NPIs and number of imported cases from 160 per day to 144 per day between March 4 and March 22. Our calibration resulted in the following estimates for the adherence to NPIs for each probability of testing value:

- Adherence level is equal to 0% between March 4 and March 11, 2020
- Adherence level increases linearly from 0% to 78% between March 12 and March 25
- Adherence level is at 85% between March 26 and April 17
- Adherence level is at 90% between April 18 and June 7
- Adherence level is at 85% between June 8 and July 1
- Adherence level is at 90% between July 2 and August 15
- Adherence level is at 80% between August 16 and September 14
- Adherence level is at 85% between September 15 and November 14
- Adherence level is at 70% between November 15 and December 5
- Adherence level is at 85% between December 6 and December 31
- Adherence level is at 80% from January 1, 2021 onwards.

Below, we first present the results of the validation, which show that COVAM was able to replicate the observed cumulative number of COVID-19 cases using the new set of values for adherence to NPIs. We then present the results of our experiments.

## Figure s5. Comparison of model predictions to actual NYC data when the baseline probability of testing is equal to 50%

Table s5. Controllable spread date and number of cases on December 31, 2021 for different daily vaccination capacity scenarios when the baseline probability of testing is equal to 50% (vaccine effectiveness 90%, vaccine coverage 50%). Numbers in parentheses represent percent reduction relative to no vaccine.

| NYC | | | | | | | | | | | | |  |
| --- | --- | --- | --- | --- | --- | --- | --- | --- | --- | --- | --- | --- | --- |
|  | 90% Adherence | | | 85% Adherence | | 80% Adherence | | 75% Adherence | | Dynamic Adherence | | | |
| Vaccination capacity | Date | Number of cases | Date | | Number of cases | Date | Number of cases | Date | Number of cases | Date | Number of cases |  |  |
| No Vaccine | 18-Mar-2021 | 699,373 | 5-Jul-2021 | | 1,003,710 | 25-Aug-2021 | 2,521,410 | 18-Jun-2021 | 3,831,060 | After June 2022 | 1,294,850 |  |  |
| 0.05% | 16-Mar-2021 | 687,666  (2%) | 3-Jun-2021 | | 925,130  (8%) | 8-Aug-2021 | 2,148,540  (15%) | 17-Jun-2021 | 3,511,630  (8%) | After June 2022 | 1,260,090  (3%) |  |  |
| 0.1% | 14-Mar-2021 | 677,418  (3%) | 15-May-2021 | | 870,559  (13%) | 22-Jul-2021 | 1,828,720  (27%) | 16-Jun-2021 | 3,190,300  (17%) | 12-Jan-2022 | 1,164,190  (10%) |  |  |
| 0.25% | 9-Mar-2021 | 652,886  (7%) | 16-Apr-2021 | | 770,915  (23%) | 4-Jun-2021 | 1,223,030  (51%) | 6-Jun-2021 | 2,263,850  (41%) | 23-Jul-2021 | 892,029  (31%) |  |  |
| 0.5% | 3-Mar-2021 | 622,681  (11%) | 24-Mar-2021 | | 685,849  (32%) | 22-Apr-2021 | 850,331  (66%) | 22-May-2021 | 1,269,030  (67%) | 1-May-2021 | 679,026  (48%) |  |  |

Table s6. Controllable spread date and number of cases on December 31, 2021 for different daily vaccination effectiveness scenarios when the baseline probability of testing is equal to 50% (vaccine coverage 50%, vaccination capacity 0.25%). Numbers in parentheses represent percent reduction relative to no vaccine.

| NYC | | | | | | | | | | |
| --- | --- | --- | --- | --- | --- | --- | --- | --- | --- | --- |
|  | 90% Adherence | | 85% Adherence | | 80% Adherence | | 75% Adherence | | Dynamic Adherence | |
| Vaccine effectiveness | Date | Number of cases | Date | Number of cases | Date | Number of cases | Date | Number of cases | Date | Number of cases |
| No Vaccine | 18-Mar-2021 | 699,373 | 5-Jul-2021 | 1,003,710 | 25-Aug-2021 | 2,521,410 | 18-Jun-2021 | 3,831,060 | After June 2022 | 1,294,850 |
| 50% | 22-Feb-2021 | 564,730 (19%) | 12-Mar-2021 | 594,723 (41%) | 18-May-2021 | 696,834  (72%) | 2-Nov-2021 | 1,218,790 (68%) | 31-May-2022 | 959,962 (26%) |
| 75% | 17-Feb-2021 | 534,767 (24%) | 26-Feb-2021 | 547,871 (45%) | 21-Mar-2021 | 577,733  (77%) | 7-Jun-2021 | 677,355 (82%) | 26-Apr-2021 | 603,508 (53%) |
| 90% | 14-Feb-2021 | 520,451 (26%) | 20-Feb-2021 | 528,286 (47%) | 4-Mar-2021 | 543,260  (78%) | 5-Apr-2021 | 580,665 (85%) | 11-Mar-2021 | 553,472 (57%) |

Figure s6. Impact of vaccine coverage on the number of confirmed cases in different regions for two different scenarios of adherence to nonpharmaceutical interventions when the baseline probability of testing is equal to 50%. In this figure, we assumed that vaccine effectiveness is equal to 90%, vaccine capacity is 0.25%/day.

a. NYC, adherence to NPIs is fixed at a level of 85%

b. NYC, adherence to NPIs is dynamic

Figure s7. Impact of vaccination and adherence to nonpharmaceutical interventions on the number of cases over time when the baseline probability of testing is equal to 50%. In these experiments, we assumed that vaccine effectiveness is 90%, vaccine coverage is 50%, and daily vaccination capacity is 0.25% for the vaccination scenario.

a. NYC, vaccination

b. NYC, no vaccination

## Section C.3 Sensitivity analysis on the modeling of vaccination effect

COVAM assumes that the vaccination is effective by simply making a proportion of the vaccinated individuals becoming immune to SARS-CoV-2 therefore do not become contagious and therefore do not spread the virus to others. For example, 90% effectiveness implies that 9 out of 10 individuals become immune and 1 out of 10 remains susceptible. An alternative way of modeling effectiveness is to assume that vaccine does not eliminate the risk of infection for vaccinated individuals but reduces the probability of these individuals becoming infected by contagious individuals. In this alternative scenario, vaccinated individuals do not become completely immune, instead, their risk of being infected is reduced by 90%. Again, we conducted this experiment only for NYC. We did not change any parameters except this structural implementation.

Below, we first present the results of the validation, which show that COVAM was able to replicate the observed cumulative number of COVID-19 cases using the new set of values for adherence to NPIs. We then present the results of our experiments.

## Figure s8. Comparison of model predictions to actual NYC data when vaccination effectiveness is modeled as a reduction in probability of becoming infected

Table s7. Controllable spread date and number of cases on December 31, 2021 for different daily vaccination capacity scenarios when vaccination effectiveness is modeled as a reduction in probability of becoming infected (vaccine effectiveness 90%, vaccine coverage 50%). Numbers in parentheses represent percent reduction relative to no vaccine.

| NYC | | | | | | | | | | | | |  |
| --- | --- | --- | --- | --- | --- | --- | --- | --- | --- | --- | --- | --- | --- |
|  | 90% Adherence | | | 85% Adherence | | 80% Adherence | | 75% Adherence | | Dynamic Adherence | | | |
| Vaccination capacity | Date | Number of cases | Date | | Number of cases | Date | Number of cases | Date | Number of cases | Date | Number of cases |  |  |
| No Vaccine | 22-Mar-2021 | 699,352 | 27-Aug-2021 | | 1,110,070 | 4-Sep-2021 | 3,307,090 | 21-Jun-2021 | 4,999,100 | After June 2022 | 1,308,990 |  |  |
| 0.05% | 20-Mar-2021 | 686,086  (2%) | 25-Jun-2021 | | 986,528  (11%) | 20-Aug-2021 | 2,764,620  (16%) | 21-Jun-2021 | 4,570,160  (9%) | After June 2022 | 1,273,190  (3%) |  |  |
| 0.1% | 17-Mar-2021 | 674,462  (4%) | 30-May-2021 | | 909,986  (18%) | 3-Aug-2021 | 2,287,610  (31%) | 19-Jun-2021 | 4,132,220  (17%) | 9-Feb-2022 | 1,193,500  (9%) |  |  |
| 0.25% | 11-Mar-2021 | 647,315  (7%) | 23-Apr-2021 | | 784,676  (29%) | 15-Jun-2021 | 1,385,800  (58%) | 11-Jun-2021 | 2,839,570  (43%) | 28-Jul-2021 | 873,254  (33%) |  |  |
| 0.5% | 3-Mar-2021 | 615,607  (12%) | 26-Mar-2021 | | 685,706  (38%) | 28-Apr-2021 | 882,795  (73%) | 5-Jun-2021 | 1,439,030  (71%) | 13-May-2021 | 689,698  (47%) |  |  |

Table s8. Controllable spread date and number of cases on December 31, 2021 for different vaccination effectiveness scenarios when vaccination effectiveness is modeled as a reduction in probability of becoming infected (vaccine coverage 50%, vaccination capacity 0.25%). Numbers in parentheses represent percent reduction relative to no vaccine.

| NYC | | | | | | | | | | |
| --- | --- | --- | --- | --- | --- | --- | --- | --- | --- | --- |
|  | 90% Adherence | | 85% Adherence | | 80% Adherence | | 75% Adherence | | Dynamic Adherence | |
| Vaccine effectiveness | Date | Number of cases | Date | Number of cases | Date | Number of cases | Date | Number of cases | Date | Number of cases |
| No Vaccine | 22-Mar-2021 | 699,352 | 27-Aug-2021 | 1,110,070 | 4-Sep-2021 | 3,307,090 | 21-Jun-2021 | 4,999,100 | After June 2022 | 1,308,990 |
| 50% | 23-Feb-2021 | 558,446 (20%) | 14-Mar-2021 | 593,368 (47%) | 13-Jun-2021 | 730,712  (78%) | 11-Jan-2022 | 1,696,450 (66%) | After June 2022 | 1,146,290 (12%) |
| 75% | 16-Feb-2021 | 527,686 (25%) | 28-Feb-2021 | 542,830 (51%) | 25-Mar-2021 | 579,748  (82%) | 30-Jul-2021 | 726,580 (85%) | 24-Jun-2021 | 629,797 (52%) |
| 90% | 14-Feb-2021 | 513,036 (27%) | 21-Feb-2021 | 521,976 (53%) | 6-Mar-2021 | 539,828  (84%) | 17-Apr-2021 | 588,620 (88%) | 21-Mar-2021 | 557,409 (57%) |

Figure s9. Impact of vaccine coverage on the number of confirmed cases in different regions for two different scenarios of adherence to nonpharmaceutical interventions when vaccination effectiveness is modeled as a reduction in probability of becoming infected. In this figure, we assumed that vaccine effectiveness is equal to 90%, vaccine capacity is 0.25%/day.

a. NYC, adherence to NPIs is fixed at a level of 85%

b. NYC, adherence to NPIs is dynamic

Figure s10. Impact of vaccination and adherence to nonpharmaceutical interventions on the number of cases over time when vaccination effectiveness is modeled as a reduction in probability of becoming infected. In these experiments, we assumed that vaccine effectiveness is 90%, vaccine coverage is 50%, and daily vaccination capacity is 0.25% for the vaccination scenario.

a. NYC, vaccinationb. NYC, no vaccination

**Supplement References**

1. Alagoz O, Sethi AK, Patterson BW, Churpek M, Safdar N. Effect of Timing of and Adherence to Social Distancing Measures on COVID-19 Burden in the United States: A Simulation Modeling Approach. Annals of internal medicine. 2020.

2. Imai N, Cori A, Dorigatti I, Baguelin M, Donnelly CA, Riley S, et al. Report 3: transmissibility of 2019-nCov. Imperial College London. Available from <https://www.imperial.ac.uk/media/imperial-college/medicine/sph/ide/gida-fellowships/Imperial-College-COVID19-transmissibility-25-01-2020.pdf>, Accessed April 30, 2020.

3. GeoDS Lab @ UW-Madison. Mapping Mobility Changes in Response to COVID-19 2021 [Available from: <https://geods.geography.wisc.edu/covid19/physical-distancing/>.

4. Google. COVID-19 Community Mobility Report 2021 [Available from: <https://www.google.com/covid19/mobility/>.

5. Unacast. Social Distancing Scoreboard 2021 [Available from: <https://www.unacast.com/covid19/social-distancing-scoreboard>.

6. United States Census Bureau. Age and Sex Table 2020 [Available from: <https://data.census.gov/cedsci/>.

7. Del Valle SY, Hyman JM, Hethcote HW, Eubank SG. Mixing patterns between age groups in social networks. Social Networks. 2007;29(4):539-54.

8. Lee BY, Brown ST, Cooley P, Potter MA, Wheaton WD, Voorhees RE, et al. Simulating school closure strategies to mitigate an influenza epidemic. Journal of Public Health Management and Practice. 2010;16(3):252-61.

9. Crisanti A, Cassone A. In one Italian town, we showed mass testing could eradicate the coronavirus. The Guardian. 2020 March 20, 2020.

10. Hu Z, Song C, Xu C, Jin G, Chen Y, Xu X, et al. Clinical characteristics of 24 asymptomatic infections with COVID-19 screened among close contacts in Nanjing, China. Science China Life Sciences. 2020;63(5):706-11.

11. CDC. Interim Clinical Guidance for Management of Patients with Confirmed Coronavirus Disease (COVID-19) 2020 [Available from: <https://www.cdc.gov/coronavirus/2019-ncov/hcp/clinical-guidance-management-patients.html#Asymptomatic>.

12. Guan W-j, Ni Z-y, Hu Y, Liang W-h, Ou C-q, He J-x, et al. Clinical characteristics of coronavirus disease 2019 in China. New England Journal of Medicine. 2020;382:1708-20.

13. Lauer SA, Grantz KH, Bi Q, Jones FK, Zheng Q, Meredith HR, et al. The incubation period of coronavirus disease 2019 (COVID-19) from publicly reported confirmed cases: estimation and application. Annals of Internal Medicine. 2020;DOI: 10.7326/M20-0504.

14. Li Q, Guan X, Wu P, Wang X, Zhou L, Tong Y, et al. Early transmission dynamics in Wuhan, China, of novel coronavirus–infected pneumonia. New England Journal of Medicine. 2020;382:1199-207.

15. Hill A. Modeling COVID-19 Spread vs Healthcare Capacity 2020 [Available from: <https://alhill.shinyapps.io/COVID19seir/>.

16. Huang C, Wang Y, Li X, Ren L, Zhao J, Hu Y, et al. Clinical features of patients infected with 2019 novel coronavirus in Wuhan, China. The Lancet. 2020;395(10223):497-506.

17. Wang D, Hu B, Hu C, Zhu F, Liu X, Zhang J, et al. Clinical characteristics of 138 hospitalized patients with 2019 novel coronavirus–infected pneumonia in Wuhan, China. JAMA. 2020;323(11):1061-9.

18. Bhatraju PK, Ghassemieh BJ, Nichols M, Kim R, Jerome KR, Nalla AK, et al. Covid-19 in critically ill patients in the Seattle region—case series. New England Journal of Medicine. 2020;DOI:10.1056/NEJMoa2004500.

19. CDC COVID-19 Response Team, Bialek S, Boundy E, Bowen V, Chow N, Cohn A, et al. Severe outcomes among patients with coronavirus disease 2019 (Covid-19)—United States, February 12–March 16, 2020. Morbidity and Mortality Weekly Report MMWR. 2020;69(12):343-6.

20. Public Health Madison & Dane County. Dane County COVID-19 Dashboard 2021 [Available from: <https://publichealthmdc.com/coronavirus/dashboard>.

21. Wisconsin Department of Health Services. COVID-19: Wisconsin Data 2021 [Available from: <https://www.dhs.wisconsin.gov/covid-19/data.htm>.

22. Czeisler MÉ, Tynan MA, Howard ME, Honeycutt S, Fulmer EB, Kidder DP, et al. Public Attitudes, Behaviors, and Beliefs Related to COVID-19, Stay-at-Home Orders, Nonessential Business Closures, and Public Health Guidance—United States, New York City, and Los Angeles, May 5–12, 2020. Morbidity and Mortality Weekly Report. 2020;69(24):751.

23. He X, Lau EH, Wu P, Deng X, Wang J, Hao X, et al. Temporal dynamics in viral shedding and transmissibility of COVID-19. Nature Medicine. 2020:1-4.

24. Li R, Pei S, Chen B, Song Y, Zhang T, Yang W, et al. Substantial undocumented infection facilitates the rapid dissemination of novel coronavirus (SARS-CoV2). Science. 2020;368(6490):489-93.

25. Liu Y, Gayle AA, Wilder-Smith A, Rocklöv J. The reproductive number of COVID-19 is higher compared to SARS coronavirus. Journal of Travel Medicine. 2020;27(2):<https://doi.org/10.1093/jtm/taaa021>.

26. Pedersen MG, Meneghini M. Quantifying undetected COVID-19 cases and effects of containment measures in Italy. ResearchGate Preprint. 2020;Available from <https://www.researchgate.net/publication/339915690_Quantifying_undetected_COVID-19_cases_and_effects_of_containment_measures_in_Italy_Predicting_phase_2_dynamics>, Accessed April 30, 2020.

27. New York City Department of Health. COVID-19: Data 2021 [Available from: <https://www1.nyc.gov/site/doh/covid/covid-19-data.page>.

28. Goodman DJ, Rothfeld M. 1 in 5 New Yorkers May Have Had Covid-19, Antibody Tests Suggest. New York Times. 2020 April 23, 2020.
