# Supplementary material for: The impact of vaccination to control COVID-19 burden in the United States: A simulation modeling approach
Source: PLoS One. 2021 Jul 14;16(7):e0254456. doi: 10.1371/journal.pone.0254456 (PMC8279349; doi:10.1371/journal.pone.0254456)
Supplement: Attachment — Submitted filename: Response to reviewers.docx [file pone.0254456.s002.docx]

**Response to Reviewers**

We thank the review team for their time and comments that have improved the manuscript significantly. We have addressed all of the comments made by the review team and incorporated suggested changes into the paper. For ease of review, we have italicized each separate question/comment from the reviewers, which are also colored in blue. Both our responses to these comments as well as changes and modifications to the original manuscript appear after the italicized reviewer comments. We believe these changes, based on the constructive and thoughtful comments of the review team, have strengthened the manuscript considerably.

**Academic Editor**

*When submitting your revision, we need you to address these additional requirements.*

*1. Please ensure that your manuscript meets PLOS ONE's style requirements, including those for file naming. The PLOS ONE style templates can be found at https://journals.plos.org/plosone/s/file?id=wjVg/PLOSOne_formatting_sample_main_body.pdf and*

*https://journals.plos.org/plosone/s/file?id=ba62/PLOSOne_formatting_sample_title_authors_affiliations.pdf*

**Response:** Done. We checked to make sure that our submission meets PLOS ONE’s style requirements.

*2. In your ethics statement in the Methods section and in the online submission form, please provide additional information about the data used in your retrospective study. Specifically, please ensure that you have discussed whether all data were fully anonymized before you accessed them and/or whether the IRB or ethics committee waived the requirement for informed consent. If patients provided informed written consent to have data from their medical records used in research, please include this information.*

**Response:** Done. We only used publicly available de-identified data for this study and stated this in the “Methods“ section.

*3. Thank you for stating the following in the Competing Interests section:*

*"OA is a paid consultant for Bristol Myers Squibb and Biovector Inc, outside of the submitted work. Other authors have no conflicts of interest to report."*

*Please confirm that this does not alter your adherence to all PLOS ONE policies on sharing data and materials, by including the following statement: "This does not alter our adherence to PLOS ONE policies on sharing data and materials.” (as detailed online in our guide for authors http://journals.plos.org/plosone/s/competing-interests). If there are restrictions on sharing of data and/or materials, please state these. Please note that we cannot proceed with consideration of your article until this information has been declared.*

**Response:** Done. We confirm that this does not alter our adherence to all PLOS ONE policies on sharing data and materials and we added the suggested statement to the manuscript.

**Reviewer #1**

*This paper examines the impact of COVID-19 vaccination in the context of current nonpharmaceutical interventions in three urban jurisdictions in the United States. A strength of their agent-based model is the detailed parameterization of interactions and representation of varying levels of adherence to NPIs. Overall, the paper is well written*

**Response:** We thank the reviewer for their time on our paper and for providing constructive comments that helped us to improve the paper.

*1. Please update the introduction to reflect the current situation with COVID-19 vaccination in the United States.*

**Response:** Done. We updated the introduction with recent vaccination information and data.

*2. In Table 1, the parameterization of vaccine effectiveness should be updated based on recent literature and data reported in real-world studies. Do you distinguish between vaccine efficacy against infection, symptomatic disease, and severe disease?*

**Response:** As presented in Table 1, we considered three different vaccine effectiveness scenarios: 50% 75% and 90%. Note that our vaccine effectiveness focuses on efficacy against infection. Recent vaccine effectiveness data show that for the three FDA-approved COVID-19 vaccines in the US, the range in Table 1 provides a reasonable range (1-5). We do not distinguish vaccine efficacy against infection, symptomatic disease, and severe disease.

*3. What is the timeline between the first and second doses? Do you account for changes in vaccine efficacy after the first and second doses, respectively?*

**Response:** This is an excellent question. Because we consider two two-dose and one single-dose vaccines, we do not explicitly model timeline between first and second doses. Instead, we assume that immunization develops after the second dose of the vaccine is administered for the two-dose vaccines. Therefore, we do not represent the changes in vaccine efficacy between first and second doses. We now clarified this in the “Methods” section of the paper as follows:

“We did not explicitly model the timeline between first and second doses for the two-dose vaccines. Instead, we assumed that immunization is developed 14 days after the second dose of the vaccine is administered for the two-dose vaccines.”

*4. The base case scenario assumes a daily vaccination capacity of 0.1% of the population per day for Dane County, Milwaukee, and NYC. Can this be supported by current vaccination roll-out data in these 3 regions? Do you anticipate this to change in the future with vaccine hesitancy?*

**Response:** The reviewer is correct that the vaccination capacity (representing the proportion of the population that is fully vaccinated in a day) changes over time. Vaccine rollout was slow in the initial weeks after the approval in December 2020, however the capacity is substantially increased since then. Recent data shows that as of April 30, 2021, approximately 45%, 36%, 38% of the population were fully vaccinated in Dane County, Milwaukee, and NYC, respectively (6-8). This corresponds to approximately a 0.25% daily capacity. Therefore, we now replaced our model’s base-case results (that had previously used 0.1% daily vaccination capacity) with 0.25% daily vaccination capacity to reflect recent vaccination data.

Vaccine hesitancy is represented in the model with a different parameter: vaccine coverage. The model distinguishes vaccination capacity and the proportion of the population who are willing to be vaccinated (vaccine coverage). As presented in Table 1, we considered a wide range of values for vaccine coverage that represents different levels of vaccine hesitancy.

*5. Does the model account for pre-existing immunity (i.e., from previous COVID-19 infection)?*

**Response:** Yes, the model accounts for pre-existing immunity from previous COVID-19 infection. We added this assumption to the “Methods” section of the paper.

*6. It is suggested in the paper that regions need to maintain a high level of adherence to NPIs to keep caseloads low. At what point in the vaccination campaign can these NPI measures be lifted successfully?*

**Response:** This is an excellent question, the answer to this question depends on the community’s behavior changes after the pandemic is over. If the community follows pre-pandemic behaviors after the NPIs are lifted, then the answer simply equals to the herd immunity levels that could be estimated by theoretical studies. In the absence of NPIs, the herd immunity levels are achieved for Dane County, Milwaukee, and NYC when 70%, 70%, and 85% of the population gains immunization through recovery from COVID-19 or vaccination. However, even after NPIs are lifted, some individuals may not follow pre-pandemic behaviors and/or continue to wear face masks therefore the timing could be earlier as these individuals will contribute to slowing of the transmission even in the absence of NPIs.

*7. A number of other agent-based modelling papers have already been published regarding the population-level impact of COVID-19 vaccination in various jurisdictions (see below). Please situate your findings in the context of the existing literature.*

*https://doi.org/10.1093/cid/ciab079*

[*https://doi.org/10.1016/j.vaccine.2021.03.058*](https://doi.org/10.1016/j.vaccine.2021.03.058)

**Response:** Thank you for pointing out these new studies, we now cited them and explained how our findings are related to these studies.

*Reviewer #2:*

*The paper presents an agent-based model integrating adherence to NPIs, vaccination strategies (and possibilities) and the interactions between both of them.*

*The current paper presents an extension of an already existing model (COVAM), adding vaccination strategies.*

*A first point is that the paper is not self-content as far as the presentation of the model is concerned: the description is too short to understand what are the characteristics and behaviors of the agents. The description makes the reader question the fact that the model is really an agent-based model and not a micro-simulation model.*

**Response:** We originally kept the model description very brief as the model was published in a prior publication. However, following the reviewer’s comment, we slightly expanded model description section with a particular focus on how agent behaviors differ. We also provide a very detailed summary of the model in the Supplement.

*p4: "The objective of this study was to use our previously developed agent-based simulation model (2),"*

*it could be clearer to express that this previously developed model is a COVID-19 model (a lot of COVID models are adaptation of models about other diseases.*

**Response:** Done.

*Figure s1. Progression of COVID-19 (adapted from Alagoz, et al 2020(1))*

*The epidemiological model has some flaws:*

*- There is no compartiment for asymptomatic, which is a key feature of the COVID-19*

*- if IM- is undetected and IM+ detected, it seems intuitive to have an arrow from IM- to IM+*

*- Instead of infected, infectious would more reflect what is important from an epidemiological point of view.*

**Response:** Figure s1 includes a compartment entitled “Exposed-incubation” in which asymptomatic individuals transmit the disease before they become symptomatic. COVAM estimates that the duration for asymptomatic infection is 2 days on average. We now stated this explicitly in the “Methods” section. IM- and IM+ are separate because IM- represents individuals who are only tested positive after their symptoms become severe enough to be hospitalized. Any IM- individual who transitions to IM+ is already included in the IM+ compartment therefore there is no arrow between the two compartments. Following the reviewer’s suggestion, we now replaced infected with infectious.

*Methods :*

*Nothing is said about experimental conditions, in particular concerning the simulations launches conditions: use of HPC or personal computers ...*

**Response:** We now explain that the code is written in C++ and we use personal computers to run the simulations.

*page9: "We ran 100 replications for each experiment" : why this number of 100 replications, how is it justified.*

**Response:** We choose 100 since the standard deviations are small enough so that we were able to obtain stable estimates for the simulations. We now added this to the “Methods” section.

*About adherence*

*"For instance, a 70% adherence level to NPIs in Dane County and Milwaukee is implemented by simply reducing the number of daily contacts from 10 per day to 3 per day per person, which slows the transmission of SARS-CoV-2." Even if an individual has a 100% adherence level to NPIs, its daily contacts will not sink to 0... (it can be in family, it will need to buy some essential goods (foods...)).*

*This idea of adherence to NPIs is interesting, but its implementation seems to simplistic.*

**Response:** The reviewer is correct and therefore the model never allows an adherence level of 100%. Because adherence is set up on a daily basis, a 100% adherence is possible for some individuals for certain days (E.g. someone living alone and not having any “close contacts”). However, in none of our simulations, we had overall adherence that reduces close interactions to 0. We used this method to implement NPIs due to its simplicity of interpretation and since it allowed us to translate cell-phone based mobility data into the adherence to NPI input.

**References**

1.	M. W. Tenforde, Effectiveness of Pfizer-BioNTech and Moderna Vaccines Against COVID-19 Among Hospitalized Adults Aged≥ 65 Years—United States, January–March 2021. *MMWR Morbidity and Mortality Weekly Report* **70**, (2021).

2.	M. G. Thompson, J. L. Burgess, A. L. Naleway, et al., Interim estimates of vaccine effectiveness of BNT162b2 and mRNA-1273 COVID-19 vaccines in preventing SARS-CoV-2 infection among health care personnel, first responders, and other essential and frontline workers—eight US locations, December 2020–March 2021. *Morbidity and Mortality Weekly Report* **70**, 495 (2021).

3.	R. A. Teran, K. A. Walblay, E. L. Shane, et al., Postvaccination SARS-CoV-2 Infections Among Skilled Nursing Facility Residents and Staff Members—Chicago, Illinois, December 2020–March 2021. *Morbidity and Mortality Weekly Report* **70**, 632 (2021).

4.	L. J. Abu-Raddad, H. Chemaitelly, A. A. Butt, Effectiveness of the BNT162b2 Covid-19 Vaccine against the B. 1.1. 7 and B. 1.351 Variants. *New England Journal of Medicine*, (2021).

5.	E. Pritchard, P. C. Matthews, N. Stoesser, et al., Impact of vaccination on SARS-CoV-2 cases in the community: a population-based study using the UK’s COVID-19 Infection Survey. *medRxiv*, (2021).

6.	New York City Department of Health. COVID-19: Data Available: <https://www1.nyc.gov/site/doh/covid/covid-19-data.page>. Accessed May 12, 2021.

7.	Public Health Madison & Dane County. Dane County COVID-19 Dashboard Available: <https://publichealthmdc.com/coronavirus/dashboard>. Accessed May 12, 2021.

8.	Wisconsin Department of Health Services. COVID-19: Wisconsin Data Available: <https://www.dhs.wisconsin.gov/covid-19/data.htm>. Accessed May 12, 2021.
